# Effects of bovine colostrum on recurrent respiratory tract infections and diarrhea in children

**DOI:** 10.1097/MD.0000000000004560

**Published:** 2016-09-16

**Authors:** Khaled Saad, Mohamed Gamil M. Abo-Elela, Khaled A. Abd El-Baseer, Ahmed E. Ahmed, Faisal-Alkhateeb Ahmad, Mostafa S. K. Tawfeek, Amira A. El-Houfey, Mohamed Diab Aboul_Khair, Ahmad M. Abdel-Salam, Amir Abo-elgheit, Heba Qubaisy, Ahmed M. Ali, Eman Abdel-Mawgoud

**Affiliations:** a Department of Pediatrics, Faculty of Medicine, Assiut University; b Department of Pediatrics, Qena Faculty of Medicine, South Valley University; c Department of Community Health Nursing, Faculty of Nursing, Assiut University; d Department of pediatrics, faculty of medicine, Al-Azhar university; e Department of Pharmaceutics and Industrial Pharmacy, Al-Azhar University, Egypt.

**Keywords:** bovine colostrum, children, diarrhea, respiratory tract infection

## Abstract

**Background:**

Bovine colostrum (BC) has direct antimicrobial and endotoxin-neutralizing effects throughout the alimentary tract, as well as other bioactivities that suppress gut inflammation and promote mucosal integrity and tissue repair under various conditions related to tissue injury. The precise role of BC in respiratory and gastrointestinal (GI) infections in children is not well defined. The aim of this study was to evaluate the efficacy and tolerability of BC administration in preventing recurrent upper respiratory tract infections (URTI) and diarrhea in children.

**Methods:**

One hundred sixty children (aged 1–6 years) having recurrent episodes of URTI or diarrhea received BC for 4 weeks. The number of episodes of URTI, diarrhea, and frequency of hospitalization required for URTI and diarrhea occurring during the study period were assessed at weeks 8 and 24.

**Results:**

From a total number of 160 children, 81 patients (50.63%) were males. The mean age (± SD) was 3.65 (± 2.01) years. The mean (± SD) total number of infections was significantly decreased after BC therapy from 8.6 ± 5.1 at baseline to 5.5 ± 1.2 after 2 months (*P* < 0.001) and to 5.7 ± 1.6 after 6 months (*P* < 0.001). The mean (± SD) total number of URTI (*P* < 0.0001), number of episodes of diarrhea (*P* < 0.001), and number of hospital admissions (*P* < 0.001) were significantly decreased after BC therapy.

**Conclusion:**

BC is effective in the prophylaxis of recurrent URTI and diarrhea as it reduces the number of episodes and the hospitalization due to these infections. Results of this study suggest that BC could be provided as a therapeutic option for children with recurrent URTI and diarrhea.

## 1 Introduction

The first human milk produced by mothers after delivery is colostrum, which is distinct in volume, appearance, and composition. Colostrum, produced in low quantities in the first few days postpartum, is rich in immunologic components such as secretory IgA, lactoferrin, leukocytes, as well as developmental factors such as epidermal growth factor. Colostrum also contains relatively low concentrations of lactose, indicating its primary functions to be immunologic and trophic rather than nutritional. Levels of sodium, chloride, and magnesium are higher and levels of potassium and calcium are lower in colostrum than later milk.^[1]^ Bovine colostrum (BC) is the first milk produced by a lactating dairy cow after the birth of the calf. Although BC is characteristically rich in many immunoglobulins to protect the neonatal bovine from environmental microorganisms, repeated immunization of a pregnant dairy cow may stimulate production of greater levels of IgG against a specified antigen.^[2–4]^ BC is homologous to human colostrum although immune factors are present in greater concentrations.^[2,3]^ This hyperimmune BC, rich in targeted IgG, is different from the conventional antimicrobials as it does not disturb the integrity of the gut microflora, nor will it potentially lead to the emergence of new antibiotic-resistant organisms.^[2,5]^ After collection, BC may be used in liquid form in its entirety, or the immunoglobulin component may be purified and fractionated into a solid concentrate. BC has been investigated as a passive immunotherapeutic agent against a wide variety of pathogens.^[2–4]^ The earliest researches of the use of BC for the treatment of gastrointestinal (GI) disease in human patients were for cryptosporidiosis. *Cryptosporidium* species generally cause self-limiting diarrhea in healthy individuals, but can cause chronic, wasting diarrhea in malnourished and immunocompromised populations.^[6,7]^ Tzipori et al^[6,7]^ described effective treatment of cryptosporidiosis in immunocompromised children and adults with BC administered orally. Using immunoglobulin concentrate derived from BC, Greenberg et al^[8]^ showed that the powdered form, but not the capsule form of the concentrate, was effective in treating diarrhea in *Cryptosporidium parvum*-infected AIDS patients.^[8]^ BC was also found effective in the prevention of cryptosporidiosis in healthy volunteers when administered before challenge with *C. parvum* spores.^[9]^ Rotavirus is the primary cause of nonbacterial, acute diarrhea in infants and young children 6 months to 2 years of age in both developed and developing countries. Mitra et al^[10]^ showed a decrease in both duration and stool output when treating rotavirus-infected infants with BC.^[10]^

BC may have direct antimicrobial and endotoxin-neutralizing effects throughout the GI tract as well as other bioactivities that decrease gut inflammation and stimulate mucosal integrity and tissue repair under various conditions related to tissue injury. Elements of BC may not only have local effects, but may also contribute to immunological events, resulting in systemic effects after contact with the gut mucosa.^[5]^ BC contains not only detectable levels of immunoglobulins far much higher (several hundred fold) than ordinary bovine milk but also contains several factors attributable to the acquired and innate immune systems. BC is a very rich source of bioactive constituents such as growth-promoting factors that act as mediators for infant growth and development in addition to a series of antimicrobial fractions including lactoferrin, lactoperoxidase, and lysozymes.^[4,5]^ BC is a rich source of growth factors, including insulin-like growth factor (IGF)-I structurally identical to that found in humans. BC contains higher concentrations of IGF-I than does human colostrum (500 compared with 18 μg/L).^[1,2]^ These growth factors are relatively stable to both heat and acidic conditions. They, therefore, survive the harsh conditions of both commercial milk processing and gastric acid to maintain their biological activity. IGF-I is known to promote protein accretion, that is, it is an anabolic agent and is responsible for mediating the growth-promoting activity of growth hormone (GH). IGF-II is present in bovine milk and colostrum at much lower concentrations than is IGF-I, but like IGF-I, it has anabolic activity and has been shown to reduce the catabolic state in starved animals.^[1,2,4,5]^

Lactoferrin is a glycoprotein with antibacterial as well as antiviral, lipopolysaccharide binding, and growth-regulating effects. Lactoperoxidase is an antibacterial enzyme that inhibits bacterial metabolism and has been shown to be toxic to a range of gram-positive and gram-negative bacteria. It also possesses antiviral activities. Lysozyme plays an important role in the innate immune system by attacking the peptidoglycan cell constituents in gram-positive bacteria, leading to bacterial lysis.^[5]^ BC contains a range of immune-regulating and inflammatory cytokines, such as interleukins (IL-1β, IL-2, IL-6, IL-17), tumor necrosis factor-α, interferon-γ, and other nonantimicrobial combinations that contribute to the control of infection and inflammation through cytokine-facilitated cross talk, pathogen recognition, and immune cell recruitment.^[5,11,12]^ In addition, specific microRNA with immune-regulating potential is present in microvesicles; it is stable under the degradative conditions of the GI tract, and may have the potential to reach the immune cells of gut-associated lymphoid tissues. Bioactive oligosaccharides may be important in protecting against pathogens and promoting the growth of beneficial microflora in the colon.^[5,11,12]^

Respiratory and diarrheal diseases are major causes of morbidity and mortality in developing countries. Recurrent GI and respiratory infections represent a serious public health problem in developing countries like our country.^[4,13]^

Acute upper respiratory tract infection (URTI) is a common disease in young children and contributes to approximately 20% of mortality in children younger than 5 years. It represents the most frequent problem in general pediatric practice and is responsible for more than a third of school absence. Most acute respiratory infections are caused by viruses and bacteria, including rhinoviruses, respiratory syncytial viruses, adenoviruses, influenza viruses, and parainfluenza viruses.^[4,13–15]^ Diarrhea on the other hand is a major killer disease in children under 5 and recurrent diarrhea affects nearly 20% of the population, thus it is an important public health problem.^[4,13]^ The World Health Organization (WHO) established the external Child Health Epidemiology Reference Group (CHERG) to develop estimates of the proportion of deaths in children younger than 5 years attributable to pneumonia, diarrhea, malaria, and measles. Of the estimated 8795 million deaths in children younger than 5 years worldwide in 2008, infectious diseases caused 68% (5970 million), with the largest percentages due to pneumonia (18%), diarrhea (15%), and malaria (8%).^[16]^

To date, the clinical trials on the use of BC in recurrent infections in children are very limited. The present study was therefore conducted to evaluate the efficacy and tolerability of BC in preventing recurrent respiratory tract infections and diarrhea in children.

## 2 Patients and methods

The Ethical Committee of Qena Faculty of Medicine, South Valley University, Egypt, approved the study. Participants were given a complete description of the study and a written informed consent in accordance with South Valley University Ethical Committee guidelines was taken from parents of all cases. The work has been carried out in accordance with the code of Ethics of the World Medical Association (Declaration of Helsinki) for experiments involving humans.

### 2.1 Study setting

The study was conducted in 2 cities in Upper Egypt. Children were from community health facilities of Assiut and Qena University Hospitals and 3 private centers form January 2015 to June 2015.

### 2.2 Study patients

In this open, multicentric, noncomparative study, a total of 160 children of either sex between 1 and 6 years of age were enrolled after obtaining informed written consent from the guardian of all subjects. Children were selected from a sample of 249 children with recurrent URTI and/or diarrhea. Recurrent URTI was defined as >6 episodes of URTI during the period of 6 months prior to enrollment in the study and recurrent diarrhea was defined as >6 episodes of diarrhea not requiring hospitalization or >2 episodes of diarrhea requiring hospitalization during the period of 6 months prior to enrollment in the study.

All study populations were in good nutritional state. All children with severe kwashiorkor, nutritional marasmus, or marasmic kwashiorkor were excluded from the study. Each family also completed a baseline dietary record, where food items consumed were classified into relevant food groups such as cereals, legumes, vegetables, meat, dairy, and fruits. This was used to calculate food diversity. A minimum of 4 food groups was considered as adequate mixture.

We excluded any subject with one or more of the following criteria: children younger than 1 year or older than 6 years; children with congenital abnormalities of respiratory and GI systems; children received immune booster supplements (e.g., *Echinacea purpurea* and *Nigella sativa*), immunomodulators, or immunosuppressive drugs such as corticosteroids; children with lactose intolerance; children with any known chronic or serious illness and primary immune deficiency diseases; and children with recent vaccination with seasonal or pandemic influenza.

Eighty-nine children were excluded from the study, 19 patients did not meet the inclusion criteria and/or have one or more of the exclusion criteria and 10 families (10 children) declined to participate in the study. Sixty children were excluded from analysis, 54 children due to loss of follow-up and 6 children due to discontinuation of the therapy; 160 children completed the trial.

### 2.3 Study design

This was a prospective, open, multicentric, cohort study in which children received BC (ImmuGuard^®^, sachets manufactured by NMI, London, England) for 4 weeks. ImmuGuard sachets contain a powdered form of the first 6 hours BC (3 g/sachet). Each sachet was added to 50 mL of neutral (previously boiled) water with continuous mixing until being dissolved. BC was advised to be taken on an empty stomach at least 30 minutes before meals. As recommended by the manufacturer, the dose of ImmuGuard is 1 sachet per day for children less than 2 years and 2 sachets per day for children older than 2 years.

### 2.4 Outcome assessment

The outcome measures were reduction in number of episodes of URTI, diarrhea, and frequency of hospitalization required for URTI and diarrhea during the study period (time from BC therapy to 6 months). Follow-up visits were done at weeks 8 and 24, and the total number of episodes of recurrent infections was assessed every 4 weeks. Each patient served as his own control, and differences at 8 and 24 weeks were calculated percentagewise using each patient's baseline value (the same period of 6 months (from January to June) in the previous year prior to enrollment as reference values). Caregivers of participating children received a weekly telephone call from the research team reminding them to administer the therapy. They were not allowed to change the provided dose or to add any supplements or pharmacotherapies throughout the study period. At follow-up visits, the parents were asked to report any difficulties or adverse effects during the study period. Side effects were recorded throughout the study and were assessed using a checklist every month.

### 2.5 Statistical analysis

Statistical Package for Social Sciences program (SPSS Inc., Chicago, IL) version 16 was used for data analysis. The data were collected, analyzed and the results were presented as mean ± standard deviation (SD). Quantitative data were analyzed using the ANOVA and Student's *t*-test. *P* value of ≤0.05 denoted the presence of statistically significant difference.

## 3 Results

The research team examined 249 children with recurrent URTI and/or diarrhea, 29 children were excluded and 220 patients enrolled to receive BC. Fifty-four patients were lost to follow-up, 6 patients withdrew due to side effects and 160 patients completed the study. The results of 160 children who completed the study were analyzed (see flow diagram). Seventy-five percent of participants were breast-fed through 12 to18 months while 25% were on bottle feeding. Table 1 shows the body mass index (BMI), clinical and demographic data of all patients. From a total number of 160 children, 81 patients (50.63%) were males and 79 patients (49.37%) were females. The mean age (± SD) was 3.65 ± 2.01 years, (range 1 to 6 years) (Table 1). The mean age of the first infection was 7 months. Table 2 shows the mean (± SD) total number of infections, URTI, diarrhea, and hospital admissions occurring during the same period of 6 months in the previous year prior to BC therapy at baseline and after 2 and 6 months of therapy. The mean (± SD) total number of infections significantly decreased after BC therapy from 8.6 ± 5.1 at baseline to 5.5 ± 1.2 after 2 months (*P* < 0.001) and to 5.7 ± 1.6 after 6 months (*P* < 0.001). The mean (± SD) number of URTI episodes significantly decreased after BC therapy from 8.2 ± 3.3 at baseline to 3.6 ± 2.2 after 2 months (*P* < 0.0001) and 3.8 ± 3.1 after 6 months (*P* < 0.0001). The mean (±SD) number of episodes of diarrhea significantly decreased after BC therapy from 6.1 ± 2.0 at baseline to 3.7 ± 2.5 after 2 months (*P* < 0.001) and 3.9 ± 2.7 after 6 months (*P* < 0.001). As regards hospital admissions and other types of infections, the patients showed significant improvements after 2 and 6 months of BC therapy. The reduction in the mean number of episodes of total number of infections, URTI, diarrhea, and hospital admissions was comparable in patients after 2 months and 6 months of therapy (Table 2). Table 3 shows the recognized etiological pathogens of diarrhea and respiratory tract infections in the study patients.

**Table 1:**
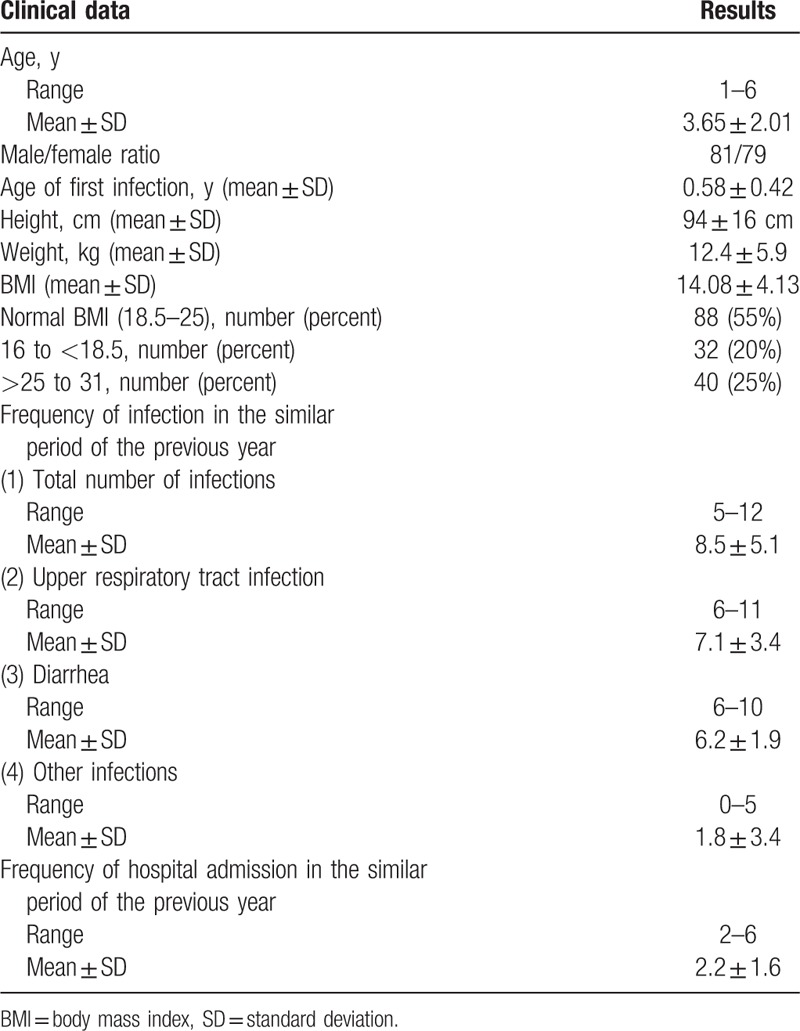
Baseline clinical data of all studied children.

**Table 2:**
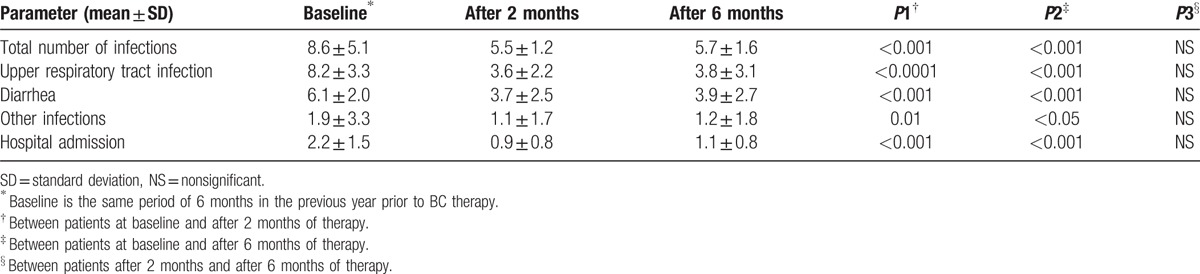
Assessment parameters at baseline and changes at 2 and 6 months after bovine colostrum therapy.

**Table 3:**
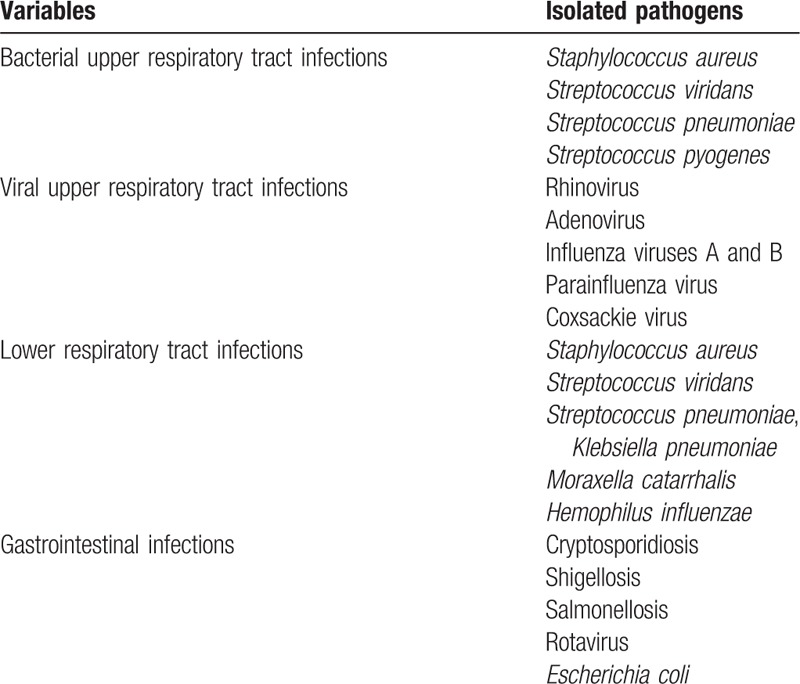
Etiological pathogens of diarrhea and respiratory tract infections.

## 4 Adverse events

BC supplementation was generally well tolerated. We reported temporally associated side effects in 12 patients (7.5%) during the study period. Skin rashes (9 patients), itching (1 patient), and diarrhea (2 patients) were reported. All side effects were mild and transient, and only 6 patients discontinued the BC treatment.

## 5 Discussion

Pediatric respiratory tract and GI infections are the most common reasons for physician visits and hospitalization, and are associated with significant morbidity and mortality.^[13]^ The diagnosis and management of recurrent respiratory tract infections in children may present a significant challenge for the primary care physician. Timing, location, and prodromes to recurrence can all provide important clues to the etiology of infection.^[4,13,15]^ Colostrum is the first natural species-specific food produced by female mammals during the first 24 to 36 hours after giving birth. Chemically, it is a very complex fluid, rich in nutrients, antibodies, and growth factors. The antimicrobial components like lactoferrin, lysozyme and lactoperoxidase, and the immunoglobulins provide passive immunity to the newborn, and the growth factors stimulate the growth of the gut.^[17]^ Whole BC and immunoglobulin-enriched colostrum (hyperimmune bovine colostrum, HBC) fractions have been used in infants and immunocompromised adults to treat or prevent infections.^[17,18]^ Knowledge on the effects of BC for treatment and prevention of infections in children is still very limited. In the present study, the number of episodes of URTI reduced significantly after 2 months of BC therapy. Also, after 6 months, the number of URTI in patients significantly reduced than baseline. Our results were in agreement with previous reports.^[3,18–21]^ Uchida et al^[19]^ demonstrated that ingestion of BC (6 to 7 days postpartum) significantly reduces the mean frequency of URTI and days of illness with fever in 3- to 6-year-old children compared with the placebo group. In a double blind, placebo-controlled, randomized trial, Brinkworth and Buckley^[20]^ retrospectively pooled data from several previous studies examining self-reported URTI symptoms in 174 healthy adult males receiving BC or a whey-protein-based control supplement. Significantly fewer subjects receiving BC reported URTI symptoms within 7 weeks after discontinuation of the intervention compared with those receiving placebo.^[20]^ In addition, Patel and Rana^[4]^ demonstrated significant decreases in URTI episodes in Indian children receiving BC. The study was conducted involving 133 pediatricians across India. Children having recurrent episodes of URTI received BC 3 g once daily for 3 months. The primary outcome measure was reduction in the number of episodes of URTI occurring during the study period (time from enrollment to 12 weeks of BC therapy) as compared with the 6 months prior to enrollment in the study. Frequency of hospitalization required for URTI during the study period as compared with 6 months prior to enrollment was also analyzed. The results of 551 patients who completed the study were analyzed. After BC therapy, the percentage reduction in the number of episodes of URTI from baseline was 73.01%, 83.25%, and 91.19% at 4, 8, and 12 weeks, respectively. Patıroğlu and Kondolot^[3]^ studied the administration of an oral lozenge containing 14 mg of colostrum and 2.2 mg of lysozyme or a placebo lozenge 3 times per day for 1 week in 31 children aged 5 to 16 years with known IgA deficiency and clinical signs of URTI. The presence of viral infection was determined clinically, and etiologies were not confirmed by laboratory investigation. No difference in serially tested salivary IgA levels was observed between groups, but 1 week of colostrum supplementation reduced infection severity scores compared with placebo. Similarly, 43.8% of patients in the colostrum group and 13.3% in the placebo group reported cessation of symptoms, but this difference was not significant.^[3]^ Jones et al^[21]^ reported significantly decreased URTI symptoms and episodes in athletes receiving BC compared with the placebo group. Cesarone et al^[22]^ reported that BC reduced flu episodes, and suggested that supplementation with BC might be more effective than vaccination against influenza virus.

Another important result of the present study was the significant reduction in the number of episodes of diarrhea after 2 months of the 4 weeks of BC therapy. After 6 months of the therapy, the number of episodes of diarrhea in patients also significantly reduced compared with baseline. In line with our results, a placebo-controlled study in 30 children affected by diarrhea caused by shiga toxin-producing *Escherichia coli* were treated with an immunoglobulin preparation extracted from normal BC and containing more than 65% immunoglobulin.^[23]^ This study showed that the BC treatment significantly reduced stool frequency, but no effects were observed on pathogen numbers or complications of infection.^[23]^ Patel and Rana^[4]^ also reported significant decreases in self-reported diarrheal episodes in Indian children receiving BC preparation for 12 weeks. Another study^[24]^ reported that the anti-rotavirus immunoglobulins of BC were effective in the management of children aged 4 to 24 months with acute rotavirus diarrhea. A daily supplement of 100 mg bovine lactoferrin for 3 months did not reduce rotavirus incidence but reduced the frequency and duration of vomiting and diarrhea episodes.^[25]^ However, no effect on diarrhea was noted in a study by Ochoa et al^[26]^, but lactoferrin supplementation significantly improved the growth of children and decreased the prevalence of *Giardia* colonization.^[26]^ Our data and the previous studies suggest that BC has a prophylactic effect against URTI and diarrhea. These data, however, are primarily self-reported and were obtained by pooling study results that were not individually significant. The studies are heterogeneous with regard to population, colostrum dosage and formulation, methodological quality, and results. Thus, this effect of BC must be confirmed in further well-designed, prospective, placebo-controlled studies.

## 6 Conclusion

In conclusion, BC is effective in the prophylaxis of recurrent URTI and diarrhea as it reduces the number of episodes and the hospitalization due to them. Results of this study suggest that BC could be provided as a therapeutic option for children with recurrent URTI and diarrhea.

## 7 Limitations of the study and impact on future research

There were some limitations of this study. First, this was an open-label study. Second, due to financial restraint, we could not investigate any assay of markers of immune functions in our patients. Further randomized, controlled, longitudinal studies with a larger sample size are warranted to confirm our findings, and to explore the mechanism of action of BC in infectious processes.
